# Effect of Fiber-Matrix Interface on the Mechanical Response of a Woven Carbon Fiber/PEEK Composite Material

**DOI:** 10.3390/ma15207340

**Published:** 2022-10-20

**Authors:** Sebastián Andrés Toro, Alvaro Ridruejo, Carlos González, Juan Pedro Fernández Blázquez

**Affiliations:** 1 Department of Materials Science, Universidad Politécnica de Madrid, ETSI Caminos, C/Profesor Aranguren, 3, 28040 Madrid, Spain; 2 Departamento de Ingeniería Mecánica, Universidad de Santiago de Chile, USACH, Av. Bernardo O’Higgins 3363, Santiago de Chile 9170022, Chile; 3 IMDEA Materials Institute, C/Eric Kandel 2, Getafe, 28906 Madrid, Spain

**Keywords:** textile composite, thermoplastic matrix, fiber/matrix adhesion, interface strength

## Abstract

This work studies the relationship between the interface shear strength (IFSS) and the mechanical response of a carbon fiber-reinforced composite with a polyether-ether-ketone (PEEK) thermoplastic matrix. Two types of laminates were studied: the first kind was manufactured with as-received fiber fabrics, while specimens belonging to the second one were fabricated with thermally treated fibers where the original sizing agent was removed. IFSS values were measured with the push-in test, showing that treated fibers exhibit a 25% higher critical shear stress. Microscopic inspection of the laminates revealed that untreated specimens were prone to debonding, generating a much higher crack density. This difference was detected by the C-Scan technique and triggered in the response of both laminates under tensile tests at ±45${}^{\circ }$ fiber orientation, where maximum stress and strain at break values of desized specimens showed an increase of 37% and 190%, respectively. Results confirmed that the original fiber sizing weakened the fiber-matrix interface. Lastly, the tensile response of the composite is analyzed in light of interface quality.

## 1. Introduction

In recent years, the sustainability of industrial processes and products has gained growing attention not only among engineers and scientists, but also investors and the public in general [1,2]. In this regard, and restricting the discussion to polymer matrix composite materials, the majority of components are manufactured with thermosetting polymers. Due to the irreversible nature of curing reactions, thermosets are difficult to recycle after the end of service life. In particular, given that they cannot be remelted and reshaped current feasible processes seek the separation and recovery of carbon fibers, which display nonetheless lower properties than virgin fibers [3]. After separation, the solid-resin content is used as a filler or energy source [4,5]. With this concern in mind, thermoplastic-based composite materials provide the most promising alternative. They have been reported to show great damage tolerance, high impact resistance and a capability to be reprocessed [6,7,8], though they have still to prove that they are fully competitive in terms of performance and reliability against well-established matrix materials, such as epoxy resins. Among thermoplastics, polyether-ether-ketone (PEEK) is a semicrystalline high-performance polymer, which stands out due to its mechanical properties, good tribological response, elevated service temperature, and chemical inertness [9,10]. Regarding architecture, 2D woven carbon fiber reinforcements (CF) present clear advantages when a balanced mechanical response along two orthogonal directions is required [11,12]. For these reasons, woven CF/PEEK laminate composites offer significant potential for application in several fields, including the aerospace and automotive industries [13,14].

When considering a new class of composite material such as CF/PEEK, load transfer from the matrix to the fiber is essential for the mechanical properties and structural integrity of the composite [15,16,17]. Load transfer is closely related to the fiber-matrix bonding and is quantified by the interfacial-shear-strength (IFSS).

Different experimental methodologies have been developed to quantify the IFSS value. In the last decades, microbonding techniques [18,19] and push-out tests [20,21] have been extensively used, but the values obtained from these tests might not be totally reliable: microbonding does not consider either the effect of surrounding fibers on the interface or that the polymer curing process in a single fiber is difficult to replicate. As far as the push-out technique is concerned, the process employed to produce specimens causes a relaxation of residual stresses which is highly relevant for thermoplastic matrix composites. As an alternative to both techniques, the push-in test stands out for being an in-situ method which is usually relatively simple to perform. This technique has been tested and verified in previously published works [22,23] and its results are easily comparable to those from push-out tests [24] to characterize the fiber-matrix interface.

Regarding interfacial properties, low wettability of carbon fibers with PEEK matrices has been reported and, hence, much effort has been made recently to improve the CF/PEEK interfacial strength through different techniques, including the design of special sizing agents [25,26,27,28] and the addition of CNTs to the surface of fibers [29,30,31]. This approach has also been used for cementitious materials [32,33]. However, it should be considered that commercial carbon fibers are usually already coated with a sizing agent, with the aim being to protect the surface of carbon fibers and help to avoid filament fluffing problems during processing, transport, and manipulation. It is in principle difficult for these agents to have a good chemical bonding with a specific thermoplastic matrix since they are generally made up of short-chain thermosetting polymers (epoxy resins or polyurethane) designed to optimize the compatibility of carbon fibers with conventional epoxy matrices. Therefore, load transfer between a PEEK-based matrix and fibers with epoxydic coating might well be less than optimal [34,35,36].

For these reasons, this study aims to examine the influence of the IFSS on a CF/PEEK woven-reinforced composite. This material was selected because it is one of the most widely studied thermoplastic polymers and because the quality of these laminates as a function of the fiber-matrix properties holds a lack of microscopic and macroscopic evidence, relating only the differences obtained in the flexural response [25,26,27,28,29,30,31].

To this end, two different CF/PEEK laminates are considered here, depending on whether they have been subjected to heat treatment to remove the sizing agent. Tensile tests performed on the woven composite show a clear improvement in the mechanical properties of specimens without coating. To study the causes of this improvement, fibers, matrix, and interface are characterized to identify which component impacts the macroscopic response. While fibers were unaffected by the removal treatment, the nanoindentation campaign shows that the mechanical properties of thermoplastic matrices in the vicinity of fibers underwent a certain degree of hardening due to an interaction with the sizing agent. Then the IFSS was obtained through the push-in technique, proving that the heat treatment has a strong influence on the interface. The results were analyzed to establish a link between matrix-fiber bonding and macroscale properties.

## 2. Materials and Methods

### 2.1. Materials

The base material for infiltration was a commercial carbon fiber woven fabric manufactured by Hexcel (HeXForce G0926). The fiber was Teijin TENAX E HTA 40, an intermediate modulus carbon fiber intended for industrial and aerospace applications. The structure of the fabric was 5H satin with a fiber areal weight equal to 375 g/m${}^{2}$. The polymeric matrix was made of PEEK, supplied as films with a thickness of 500 μm by Bieglo GmbH (Hamburg, Germany).

### 2.2. Heat Treatment of Dry Fabrics

**Figure 1:**
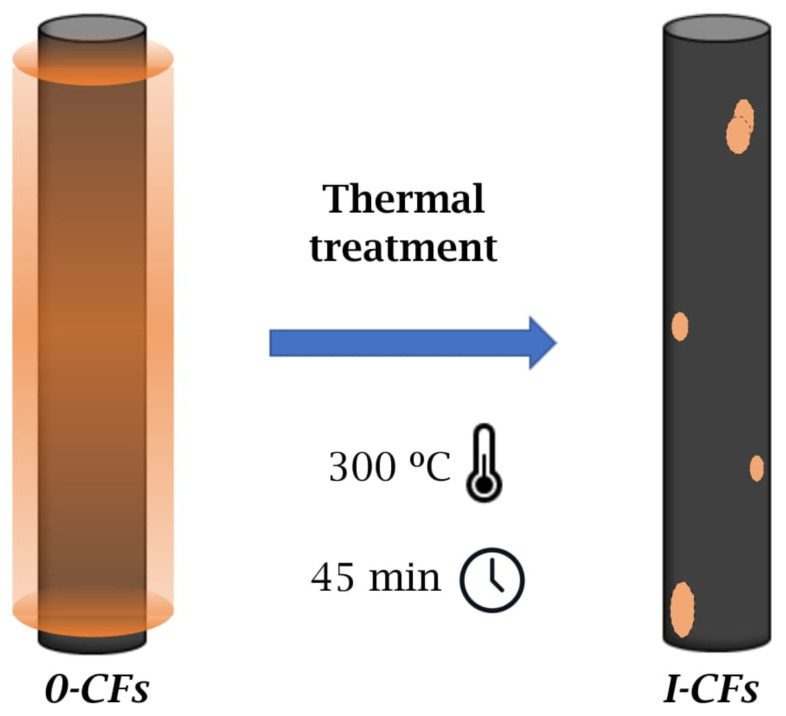
Schematic of thermal treatment. Commercial fibers and fabrics are classified as *0-CFs* (untreated) or *I-CFs* (thermally treated).

This treatment constitutes a critical step. It is applied to remove the sizing agent and therefore modifies the fiber surface, subsequently altering the fiber-matrix interface. Woven fabrics were heated in an oven for 45 min at 300 ${}^{\circ }$C to remove the sizing agent. In addition to this, fabrics were washed and rinsed with distilled water to ensure the complete elimination of residues and dried in a furnace for ten hours at 80 ${}^{\circ }$C. After the treatment described above, the fabrics underwent a measured weight loss of about 3%, still being fully manipulable. Henceforth, as-received fabrics and thermally treated ones are labeled as *0-CF* and *I-CF*, respectively. A representative diagram is shown in Figure 1.

### 2.3. Manufacturing of Composites

**Figure 2:**
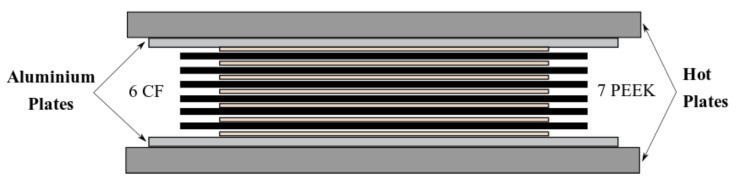
Multilayer setup for the laminate consolidation with a hot plate press.

For proper manufacturing, six square sheets of dry fabric, with dimensions 270 mm × 270 mm, were symmetrically stacked on the sides of the fabric of exposed warp yarn facing each other at the midplane and parallel to the longitudinal direction. Seven PEEK films of 210 × 210 mm were stacked between fabrics to produce an alternated dry fabric—PEEK multilayer, as shown in Figure 2.

**Figure 3:**
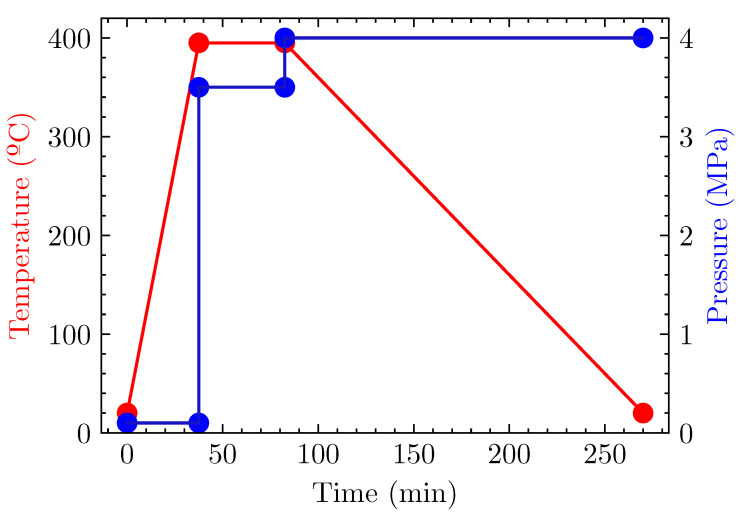
Consolidation process. Temperature (red) and pressure (blue) are plotted vs. time.

Laminates were consolidated under controlled temperature and pressure with a Fontijne Press TP400 hot press, which has a maximum capacity of 400 kN and a maximum service temperature of 400 ${}^{\circ }$C. The processing parameters are depicted in Figure 3. Starting at room temperature, a heating rate equal to 10 ${}^{\circ }$C/min was set until the dwell temperature was reached. The consolidation was made at 395 ${}^{\circ }$C under a pressure of 3.5 MPa for 45 min. Lastly, keeping a pressure of 4 MPa, a cooling rate of 2 ${}^{\circ }$C/min was prescribed. Such a slow rate promoted a high degree of crystallinity [37]. All specimens had thickness values in the range of 1.9–2.0 mm.

In order to compare similar materials, the consolidation cycle depicted in Figure 3 was the same for laminates with untreated (*0-CFs*) and treated (*I-CF*) dry fabrics. With the aim of keeping the notation, the corresponding laminates with untreated and treated fabrics were labeled as *Lam${}_{0}$* and *Lam${}_{I}$*, respectively.

### 2.4. Experimental Methods

The surface morphology of carbon fibers (CFs) was studied with a Zeiss Gemini scanning electron microscope (SEM) at 3.0 keV acceleration voltage. To better understand the chemical effects of the thermal treatment on the surface of fibers, X-ray Photoelectron (XPS) spectroscopy measurements on treated and untreated surfaces were performed in a device (PECS GmbH) equipped with a PHOIBOS 150 9MCD analyzer. The excitation source used was nonmonochromatic Mg, located at 55${}^{\circ }$ relative to the analyzer axis and operated at 200 W and 12 kV. Data processing was performed with commercial software (Casa Software Ltd., Teignmouth, UK). All elements present in the samples were identified from survey spectra (acquired at a pass energy of 50 eV). To obtain more detailed information about the chemical structure, C-1s, O-1s, and N-1s high-resolution spectra were recorded at 20 eV pass energy. The atomic concentration of the detected elements was calculated by using the integral peak intensities and the instrument sensitivity factors supplied by the manufacturer.

With the objective of analyzing possible variations in the mechanical properties of thermally treated fibers, tensile tests on the monofilaments of carbon fiber were carried out according to ASTM standard D3379. These filaments were randomly extracted from fiber bundles. The testing machine used was a Textechno Favimat+ model with a 210 cN load cell and pneumatic grips. Tests were carried out at room temperature, at a crosshead speed of 1 mm/min, with an initial gauge length of 20 mm. Excluding those cases where the fiber slipped or failed near the grips, 40 samples were tested. For statistical analysis, the failure load values were fitted to a Weibull distribution.

Thermogravimetric analysis (TGA) measurements were carried out on specimens (2–3 mg in weight) extracted from carbon fiber bundles at a heating rate of 10 ${}^{\circ }$C/min in air atmosphere using a thermogravimetric analyzer (TA Instruments Q50). Untreated (*0-CFs*) and thermally treated (*I-CFs*) fabrics were subjected to TGA under a constant flow rate of 5 mL/min. The thermal decomposition of each sample was studied within a temperature range of 50–1000 ${}^{\circ }$C. Weight loss and temperature were recorded and analyzed.

In-situ nanoindentation tests on CF-reinforced PEEK matrix and ex-situ ones on neat PEEK were performed with a NanoTest machine from Micromaterials Ltd. (Wrexham, UK). Load-controlled nanoindentation tests, up to 7 mN, were carried out with an exponential loading ramp to obtain a constant strain rate of 0.04 s${}^{-1}$ [38], with a dwell time at maximum load of 20 s and a fixed time of unloading ramp of 2 s. All indentation tests were performed with a Berkovich tip. Ten indentations on the matrix were carried out in a resin pocket with a radius of at least 20 times greater than the maximum indentation depth for each kind of matrix, as recommended in [39]. Regarding ex-situ tests, ten indentations were performed on 20 × 20 mm${}^{2}$ square samples obtained from neat PEEK films. The elastic modulus *E* and hardness *H* data were extracted from the load *vs.* displacement curve by using the Oliver-Pharr method [40].

Interface strength is measured by means of the fiber push-in technique described and validated in previously published works [22,23]. According to the shear-lag model [41] and the description of the local environment factor [42], the maximum shear stress at the onset of debonding $\tau_{c}$ can be assimilated to the IFSS as follows:

(1) $$\tau_{c}=\frac{S_{0}P_{c}}{2\pi^{2}r_{f}^{3}E_{f}},$$

where $S_{0}$ represents the slope of the linear region of the load (*P*)-displacement (*h*) curve, $P_{c}$ is the critical load value required to trigger the debonding associated with the onset of nonlinearity in the *P*-*h* curve, $r_{f}$ is the fiber radius and $E_{f}$ corresponds to the longitudinal elastic modulus of fibers. Small sections of laminates were embedded in an epoxy mount, polished sequentially with P320, P600, P1200, and P2000 alumina paper, and finally with 6, 3, and 1 μm diamond polishing paste. All tests were carried out in a Hysitron TI 950 Triboindenter equipped with a flat conical diamond tip of 5 μm in diameter; eight tests under a controlled displacement of 50 nm/s and a fixed penetration depth of 800 nm for each kind of laminate were performed to compare the values of $\tau_{c}$.

The cross-section of laminates was studied through optical microscopy. The images were acquired with an Olympus BX51 microscope. The same methodology described in Section 2.4 was adopted to grind and polish the samples. The images were used to confirm that the polymer had correctly infiltrated the fabric and measure the distributed crack density inside tows after processing [43].

Non-destructive C-Scan inspections of the laminates were carried out with a TecniTest equipment model Triton 1500, which works with a 5 MHz pulse-receiver flat transducer immersed in water to detect defects (acoustic impedance discontinuities) in the laminate. The transducer had a diameter of 9.52 mm and a focal length of 33 mm.

**Figure 4:**
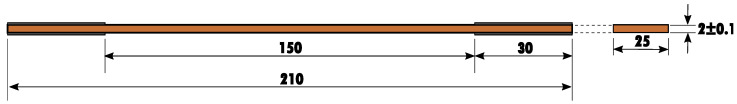
Technical drawing of a tensile specimen. Dimensions in mm.

Tensile tests were performed according to ASTM 3039 [44] (Figure 4), with a 50 kN Instron universal testing machine (Instron 8501, Instron, Norwood, MA, USA).

**Figure 5:**
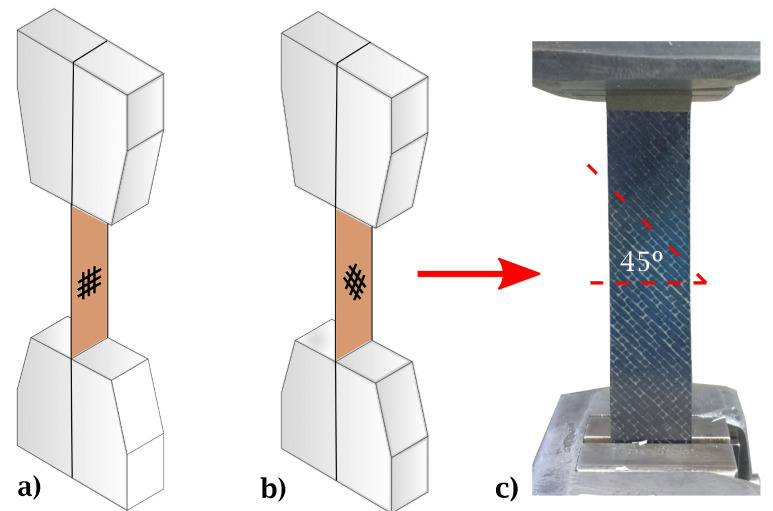
Schematic representation of tensile tests for fiber reinforcement configurations at (**a**) 0${}^{\circ }$/90${}^{\circ }$ and (**b**) ±45${}^{\circ }$ with respect to loading direction. (**c**) Experimental setup (reinforcement at ±45${}^{\circ }$).

Five specimens for each case were placed and tested in ±45${}^{\circ }$ and 0${}^{\circ }$/90${}^{\circ }$ fiber orientations with respect to the load direction as shown in Figure 5. Abrasive material in the grips was employed to prevent slippage.

## 3. Results

### 3.1. Fibers Analysis

**Figure 6:**
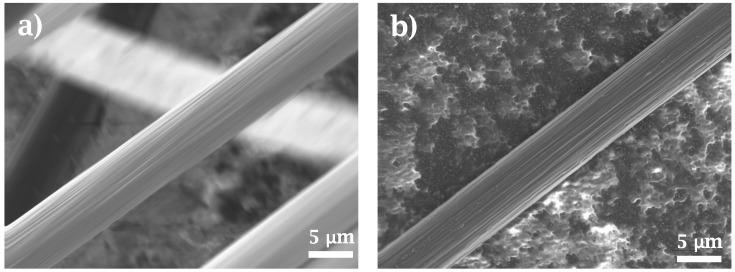
SEM images for (**a**) untreated (*0-CFs*) and (**b**) treated fibers (*I-CFs*). Surface roughness is partially masked by the sizing agent in the case of untreated fibers.

Surface morphology is shown in Figure 6 for untreated and thermally treated fibers. In both cases, the surface texture consists of long, narrow grooves parallel to the fiber axis. Roughness is mildly attenuated in untreated specimens by the presence of the coating agent. There is no evidence of surface degradation after the thermal treatment reported here.

The chemical composition of the surface of carbon fibers was examined by means of XPS. Table 1 shows the concentration of the most representative elements on both kinds of carbon fibers. Atomic contents of carbon, oxygen, and nitrogen in untreated fibers were 78.4%, 20.5%, and 1.1%, respectively, with an O/C ratio equal to 0.26. Treated fibers present a similar composition, excepting a slight decrease in nitrogen, which is linked to a lower presence of the sizing agent.

**Figure 7:**
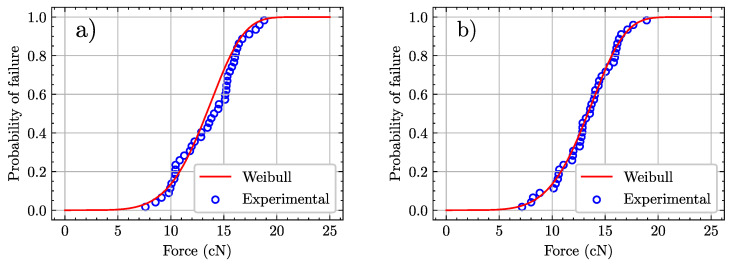
Cumulative failure distribution as a function of load for (**a**) untreated (*0-CFs*) and (**b**) trated (*I-CFs*) monofilaments. Experimental data are plotted as discrete points and fitted to a Weibull distribution (red line).

To assess the effect of thermal treatment on fibers, the failure load of CFs is measured for untreated and treated samples. Strength is measured in terms of load instead of stress because sizing is not uniform and leads to a large scatter in the values of fiber diameter and fiber mass per unit length, with a negligible contribution to mechanical properties. By contrast, the maximum (failure) load provides a stable and straightforward comparison. Fiber strength can be accordingly described by the following Weibull distribution:

(2) $$P_{b}=1-exp\left[-{\left(\frac{F_{b}}{\beta }\right)}^{\alpha }\right],$$

where $P_{b}$ is the failure probability, $F_{b}$ is the failure load, α is the shape parameter, and β is the scale parameter (see [45] for a more detailed description of these parameters). The experimental probability of failure is given by

(3) $$P_{b}=\frac{i-0.3}{n+0.4},$$

where *i* is the sample number in ascending order of strength, and *n* is the total number of tested samples. The comparison between experimental data by using Equation (2) and the fitted Weibull distributions are presented in Figure 7, for both kinds of CFs. A good fit was obtained in all cases.

The average value of the failure load, together with the respective parameters obtained from the fitted Weibull distribution, are displayed in Table 2.

These results confirm that the mechanical properties of fibers are unaffected by the heat treatment since the average strength values and statistical parameters of treated fibers are similar to those of untreated ones. This is consistent with the results reported in [35], where fibers did not show a significant decrease in strength after a treatment up to 300 ${}^{\circ }$C. In addition to this, it is worth noting that similar values of the shape parameter α indicate that both sets of fibers display similar strength variability (this arises from comparable flaw size distributions).

**Figure 8:**
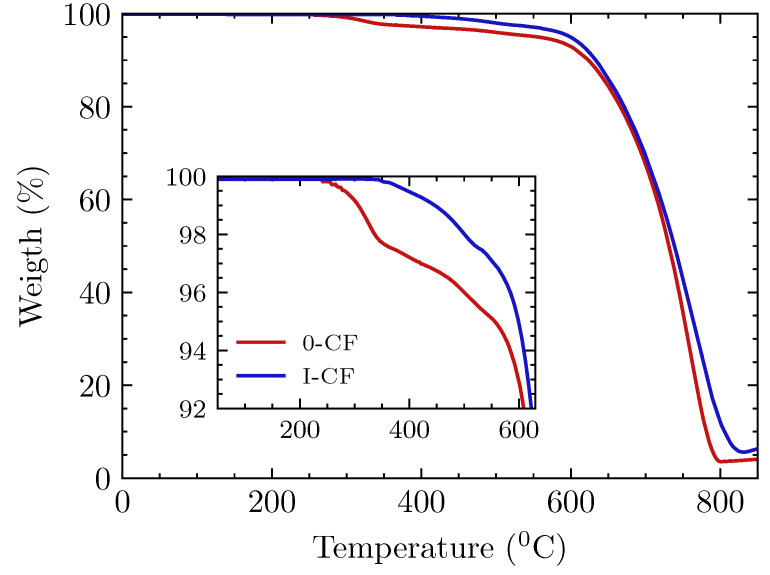
TGA analysis of untreated carbon fibre (*0-CFs*) and treated ones (*I-CFs*). Inset: untreated specimens underwent weight loss from 280 ${}^{\circ }$C onwards due to sizing decomposition.

The thermal stability of carbon fibers was analyzed by means of thermogravimetric analysis (TGA). Experiments were performed from 25 ${}^{\circ }$C to 900 ${}^{\circ }$C in air atmosphere. Weight variations for carbon fibers with and without treatment are shown in Figure 8. Regardless of whether fibers had been treated or not, the onset of carbon fiber degradation occurred at a similar temperature, around 610 ${}^{\circ }$C, followed by a similar degradation process, which implies that the core of carbon fibers remained unaltered after treatment. However, important differences are found at lower temperatures: untreated fibers showed a weight loss of 4.5% from 280 ${}^{\circ }$C to 550 ${}^{\circ }$C, due to the decomposition of sizing. The effect of heat treatment is best observed in this sizing degradation. In the case of fibers subjected to thermal treatment, the onset of decomposition was delayed until 375 ${}^{\circ }$C and the total weight loss was 2.5%.

### 3.2. Matrix Characterization: In-Situ and Ex-Situ PEEK Nanoindentation

**Figure 9:**
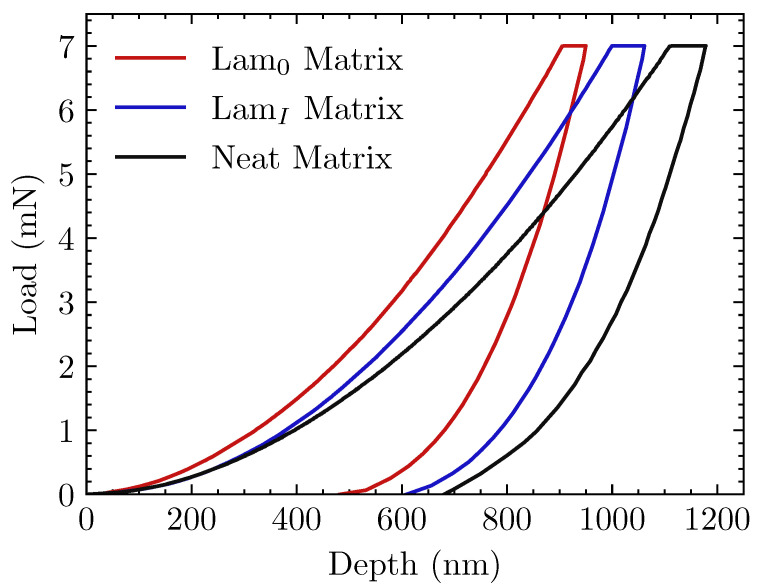
Representative load–depth nanoindentation curves for each matrix. Ex-situ curve on the neat matrix is provided for contrast.

**Figure 10:**
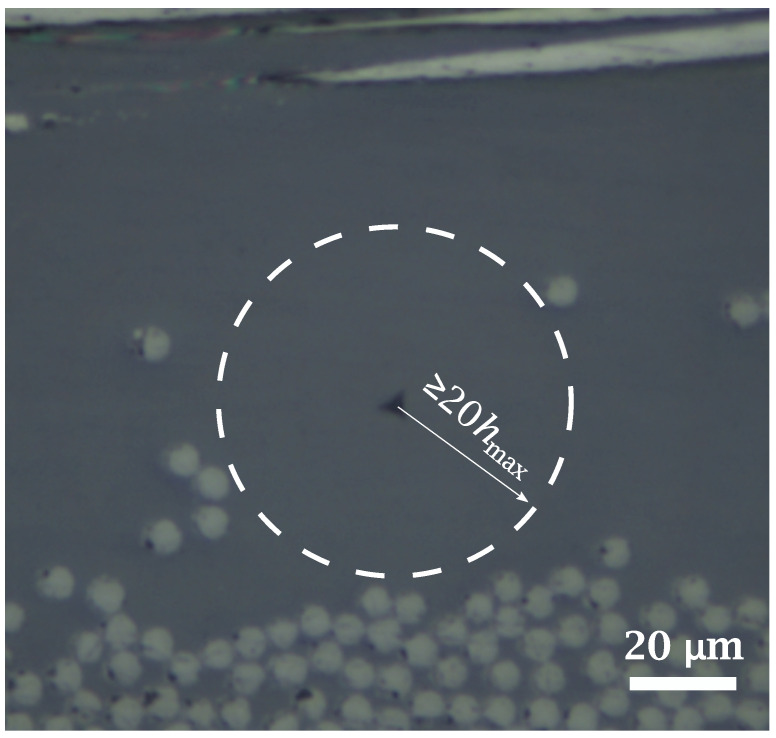
Image of an in situ indentation on PEEK matrix. Indentations were performed on resin pockets (marked by dashed line) to avoid the influence of surrounding fibers.

Representative load–penetration depth (P−h) curves are shown in Figure 9 and a corresponding image of the PEEK matrix surface after indentation in Figure 10.

Indentation depths between 850 nm and 1200 nm (depicted in Figure 9) were reached in all tests to reduce experimental scatter due to the so-called “bimodal behavior" of this semicrystalline material, as reported in [46]. For this range of indentation depth, and according to the recommendations described in [39], the resin pocket associated with in-situ inspections should have a radius of at least 20 μm, as shown in Figure 10. Hardness (*H*) and elastic modulus (*E*) of the ex-situ and in-situ PEEK specimens, calculated by the Oliver–Pharr method, are listed in Table 3.

According to Figure 9, obtained with a maximum indentation load equal to 7 mN, there is a clearly noticeable difference between in-situ and ex-situ nanoindentation responses. Previous works on neat PEEK nanoindentation [47] report average values around 4.8 GPa for elastic modulus and 0.35 GPa for hardness. Results in Table 3 confirm that the as-received PEEK properties in this work are similar to those found in the aforementioned references. As can be seen in Table 3, both elastic modulus and hardness values corresponding to in-situ tests (treated and untreated) are much higher compared with the ex-situ ones on neat PEEK. This is related to the manufacturing process of the laminates, in which the consolidation cycle promoted a higher level of crystallinity due to heterogeneous nucleation (PEEK crystallites on carbon fiber) and the slow cooling of the polymer matrix after consolidation.

Restricting the study to in-situ results, the P−h curves corresponding to *Lam${}_{0}$* (laminates made with untreated reinforcement) reached less penetration depth (see Figure 9), which suggested that the PEEK matrix was stiffer when the sizing agent was present. The average values for both hardness and elastic modulus in Table 3 are also higher for untreated specimens. This phenomenon is attributed to the fact that during the consolidation process, the sizing agent can diffuse throughout the matrix. Since PEEK remains in a liquid state for a significant time during consolidation, long-distance diffusion is greatly facilitated.

### 3.3. Push-In Test

**Figure 11:**
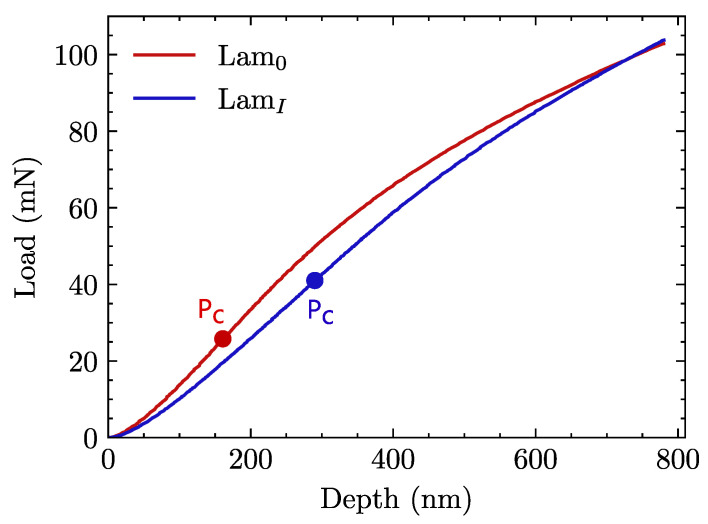
Push-in test: representative P−h curves for untreated (*Lam${}_{0}$*) and treated laminates (*Lam${}_{I}$*). Critical load, $P_{c}$, is indicated for each case.

Results of push-in tests are plotted in P−h coordinates. Representative curves corresponding to laminates *Lam${}_{0}$* and *Lam${}_{I}$* are shown in Figure 11. Only one curve is plotted for each case due to the good repeatability of experiments. The measured fiber radius values $r_{f}$, as well as the calculated parameters of the critical load $P_{c}$, maximum slope $S_{0}$, and critical interface shear strength $\tau_{c}$, are shown in Table 4.

Reaching the critical load implies a relative displacement between fiber and matrix. As can be observed, debonding in untreated (*Lam${}_{0}$*) specimens appears at a significantly lower depth and strength if compared with those where sizing had been removed (*Lam${}_{I}$*). Taking into account that the same reference values provided by the manufacturer for the elastic modulus of fibers (240 GPa) have been considered for both cases, that only fibers with similar dimensions have been tested, and that slope ($S_{0}$) values are very similar between *Lam${}_{0}$* and *Lam${}_{I}$*, it is clear that the critical load value $P_{c}$ (see Equation (1)) controls the interfacial shear stress for each case. Given that the value of $\tau_{c}$ was approximately 24% higher for laminates made with thermally treated fibers (*Lam${}_{I}$*), the quality of the PEEK/desized fibers interfaces significantly improved with respect to untreated ones.

### 3.4. Laminate Inspection

Composite laminates were inspected with an optical microscope to analyze the quality of polymer impregnation in the textile and the occurrence of defects such as voids or cracks.

**Figure 12:**
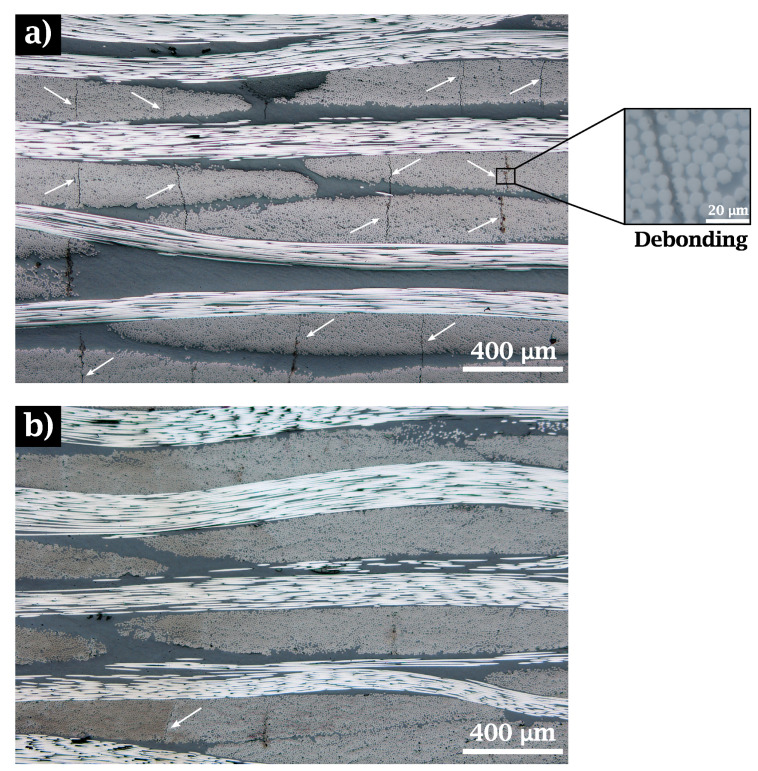
Cross section of laminates for (**a**) Untreated (*Lam${}_{0}$*) and (**b**) treated (*Lam${}_{I}$*) composites after consolidation. White arrows show initial debonding.

As can be seen in Figure 12a,b, a high volumetric fraction (close to 70%) is achieved in the tows for both cases. Very low porosity was also observed in both untreated and treated specimens. Considering that residual stresses may appear during the cooling stage due to the significant mismatch of the fiber and matrix thermal expansion coefficients [48,49,50] and as these stresses can be large enough to cause interfacial damage [51,52], it is clear from the measurements of $\tau_{c}$ (Table 4) that the debonding phenomena, and therefore the propagation, should be significantly reduced in *Lam${}_{I}$* samples. To verify this hypothesis, the distance between two neighboring cracks for both laminates was used to quantify the density of transversal cracking. Average values of 2.59 mm${}^{-1}$ and 0.25 mm${}^{-1}$ were measured for *Lam${}_{0}$* and *Lam${}_{I}$*, respectively. This provides additional evidence that fibers where the original sizing has been removed exhibit a stronger interface with PEEK.

**Figure 13:**
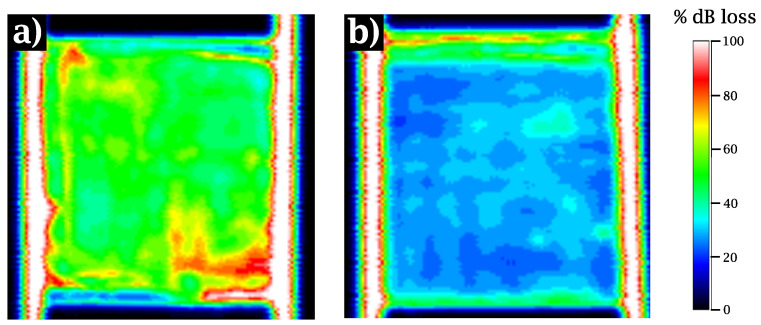
C-Scan inspections for (**a**) Laminates with *0-CFs* (*Lam${}_{0}$*), and (**b**) Laminates with *I-CFs* (*Lam${}_{I}$*). The color bar indicates wave intensity attenuation.

With the aim of evaluating further the quality of the laminates as a whole, Figure 13 provides the attenuation of ultrasound waves. The color scale ranges of attenuation from black, which implies full reflection and total impregnation of the composite, to white (total wave attenuation), which in turn indicates local decohesion, a large population of defects or porosity corresponding to poor impregnation zones [53,54].

In general, both kinds of specimens were of acceptable quality, but the attenuation of untreated laminates was stronger. Figure 13a, shows that the returned wave intensity for untreated laminates was on average 50% of the incident one. In contrast, as can be seen in Figure 13b, the returned wave intensity for their treated counterparts reached almost 70%. This result reveals that the untreated laminate presents a larger amount of distributed defects that scatter the ultrasonic wave. This is related to a poorer matrix-fiber interface, which promoted small-scale debonding and the consequent nucleation of the cracks observed in the micrographs.

### 3.5. Tensile Tests on Laminates

**Figure 14:**
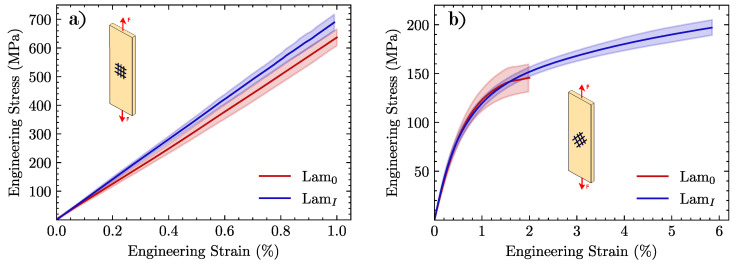
Stress-Strain curves comparison of the different CF/PEEK laminates tested in (**a**) 0${}^{\circ }$/90${}^{\circ }$ and (**b**) ±45${}^{\circ }$. Specimens were tested up to failure.

The mechanical behavior of the different specimens subjected to the tensile test is shown in Figure 14 by plotting the engineering stress-strain curves of laminates subjected to a tensile load in 0${}^{\circ }$/90${}^{\circ }$ and ±45${}^{\circ }$ with respect to the fibers. Table 5 displays the elastic modulus *E*, failure stress $\sigma_{f}$ and strain to failure $\epsilon_{f}$.

In tensile tests at 0${}^{\circ }$/90${}^{\circ }$ (Figure 14a), there were no significant differences between untreated and desized specimens. Very similar values of strength and strain to failure were found: 640 MPa and 1.0% final values for *Lam${}_{0}$* and 690 MPa and 0.99% for *Lam${}_{I}$*. Since in the 0${}^{\circ }$ direction, fibers essentially carry all load in the laminate, neither the matrix nor the interface is expected to play a relevant role. However, for those specimens tested at ±45${}^{\circ }$ orientation, a dramatic extension of the curve in the plastic regime was observed for desized (*Lam${}_{I}$*) specimens, as compared to *Lam${}_{0}$*. With an increase of 24% in the $\tau_{c}$ value reported in the push-in test, strength ($\sigma_{f}$) and strain to failure ($\epsilon_{f}$) rose by 37% and 190%, respectively. This test provides evidence that there is a critical influence of the fiber-matrix bonding quality on the behavior of the composite.

**Figure 15:**
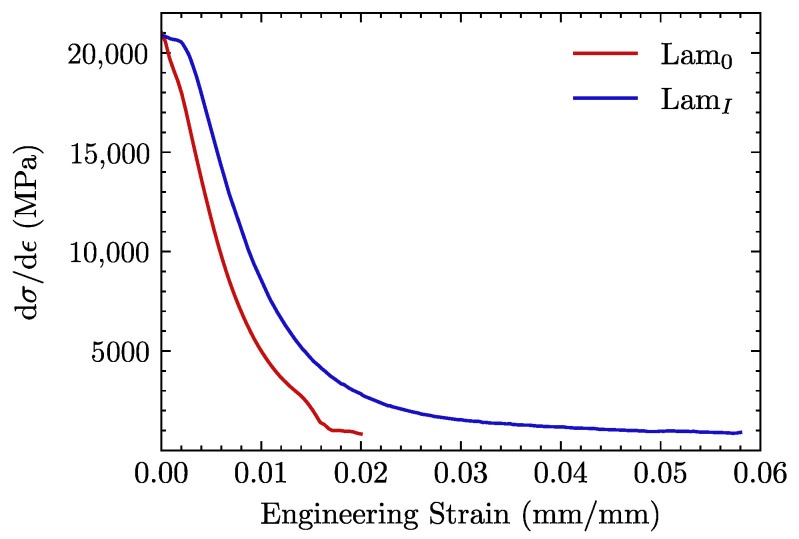
Stress hardening rate dσ/dϵ vs strain for *Lam${}_{0}$* and *Lam${}_{I}$* specimens subjected to tensile loading with woven reinforcement at ±45${}^{\circ }$.

Besides the extension of the curve reported for ±45${}^{\circ }$ tests, it is interesting to plot its derivative dσ/dϵ, which represents the tangent stiffness of the material. As can be observed in Figure 15, *Lam${}_{0}$* specimens display an earlier drop in the dσ/dϵ curve related to the poorer fiber-matrix load transfer. There is a secondary drop prior to failure in the region ϵ≈0.015, which is likely caused by the propagation of preexisting cracks.

### 3.6. Post-Fracture Inspection

**Figure 16:**
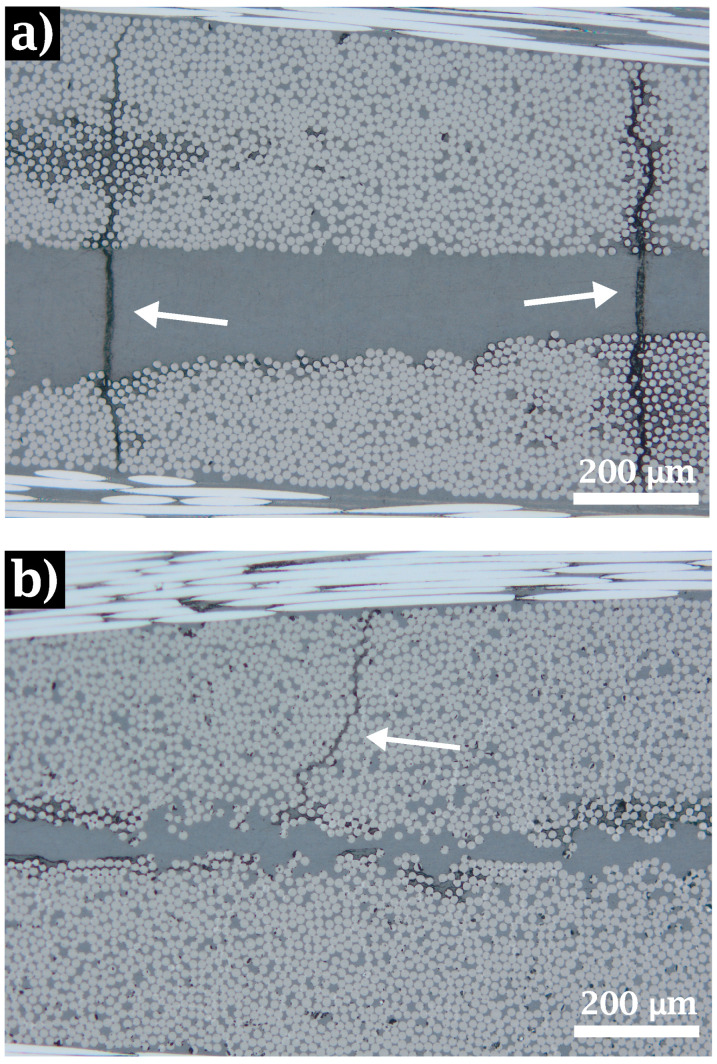
Images of the laminate cross-section (post-mortem). The samples obtained corresponds to different materials: (**a**) *Lam${}_{0}$*±45${}^{\circ }$ and (**b**) *Lam${}_{I}$*±45${}^{\circ }$. White arrows show the cracking pattern.

To analyze the influence of the heat treatment on the laminate failure, several cross-sectional images were taken at approximately 2 cm from the fracture line of the specimen, considering that at this distance the material undergoes damage similar to that occurring in the breakage zone. These samples were obtained from the postmortem tensile specimens with fiber reinforcement oriented at ±45${}^{\circ }$. Figure 16 shows the cracking pattern in the mentioned cross-section.

For untreated (*Lam${}_{0}$*) samples, extensive crack propagation can be observed. These cracks pass through the fiber bundles and the resin pockets (see Figure 16a. In treated (*Lam${}_{I}$*) composites, the improved values of interface strength prevent the crack from propagating across the tows, as shown in Figure 16b.

## 4. Discussion

Results prove that there is a noticeable effect of fiber-matrix interfacial shear strength (IFSS) on the mechanical properties of a CF/PEEK composite, particularly when reinforcement orientation causes the load to be carried to a greater extent by the matrix.

If the main results are revisited in terms of the relevant factors (properties of fibers, matrix, and interface), the first conclusion, regarding fibers only, is that the thermal treatment performed on the fabrics to eliminate the sizing agent does not affect the mechanical performance of individual fibers, as shown by Weibull statistics.

In terms of the chemical analysis on the surface, it is generally believed that the O/C ratio is a quantitative measurement of oxygen-containing functional groups on carbon fibers’ surface and a good indicator of the effective surface area of chemical bonding between carbon fibers and resins [55,56]. In the case of this study, this ratio evolved from 0.25 to 0.24, suggesting that the surface activity remained unchanged. The reduction in nitrogen content after the treatment and the mass loss in TGA analysis further confirms that the heat treatment decreased the amount of the sizing agent without causing any other effect on carbon fibers.

Regarding the in-situ micromechanical characterization of the PEEK matrix, a noticeable hardening of laminates made of untreated specimens (*Lam${}_{0}$*) can be observed in Table 3, as compared with those made of treated ones (*Lam${}_{I}$*). As the sizing agent present in *Lam${}_{0}$* underwent degradation (when T > 280 ${}^{\circ }$C) during the manufacturing process in the press, its residual products diffused into molten PEEK and induced matrix stiffening. Nonetheless, given that load is carried primarily by fiber reinforcement, this hardening effect in the matrix does not have a significant impact on the properties of the composite laminates at the macroscopic scale.

At the micromechanical level, the property which exhibited the most marked differences depending on whether the laminate was treated or not was the IFSS, quantified as the value of $\tau_{c}$ measured with the push-in experimental technique. Here, the value of $\tau_{c}$ increased by 25% with respect to the untreated composites. It is clear that the PEEK matrix does not create strong chemical bonds with sizing agents designed for thermosets in general and for epoxy resins in particular [35,57]. This weak bonding has a decisive impact on CF/PEEK composites at the mesomechanical and macromechanical scales. Firstly, cracks appear where a weak interface is unable to withstand the stresses originated by thermal effects, as shown in Figure 12. The debonding phenomenon observed between fiber and matrix is apparent in *Lam${}_{0}$*, with a crack density ten times as high as that recorded in *Lam${}_{I}$*. Secondly, ultrasonic inspection confirms that laminates with a more robust interface present a much smaller number of imperfections thanks to better matrix-fiber adhesion.

Tensile tests with fiber reinforcement oriented at ±45${}^{\circ }$ are arguably the most conclusive ones at the macro-scale since they critically depend on the fiber-matrix interfacial strength. As shown in Figure 14b, there is a remarkable improvement in the capability of the desized material to deform in the plastic regime. At 1% strain, a difference in the hardening curves is already visible in Figure 15. After this point, untreated (*Lam${}_{0}$*) specimens undergo an early localization of damage leading to failure at a strain close to 1%. This difference could be attributed to the mechanical behavior of the CF/PEEK laminates to the initial state of the *Lam${}_{0}$* specimens. A large number of cracks were observed in this material, caused by the debonding between the matrix and the fiber prior to the mechanical tests. This initial state triggered an accelerated localization of the damage that is particularly relevant when the specimen is subjected to the ±45${}^{\circ }$ tensile test. This effect could be related to the observations made in the postmortem specimens of the tests at ±45${}^{\circ }$, in which the original crack pattern is observed to grow in *Lam${}_{0}$* samples, in contrast with laminates with desized reinforcement (*Lam${}_{I}$*), where the enhanced fiber-matrix interaction hinders crack propagation until high stress levels are reached. The tensile response of the thermally treated (*Lam${}_{I}$*) material shows a robust performance. Indeed, previous published studies with woven reinforcement for epoxy matrices reported very similar responses for tests at 0${}^{\circ }$/90${}^{\circ }$[58,59] and ±45${}^{\circ }$ fiber orientation [60,61].

## 5. Conclusions

The main conclusions of this work are summarized below:

- The thermal treatment applied to the fibers (45 min at 300 ${}^{\circ }$C in ordinary air atmosphere, followed by washing and drying) was able to remove most of the epoxydic fiber coating present in the original woven carbon fiber reinforcement. Total removal was not achieved and no significant chemical changes were detected.
- Carbon fibers were undamaged by the thermal treatment
- In untreated specimens, a degradated form of the sizing agent diffused into molten PEEK, causing a noticeable hardening of the final matrix (above 30% with respect to reported values of elastic modulus of the neat resin)
- Push-in tests showed that the interfacial shear strength of thermally treated specimens increased by 24% compared with their untreated counterparts
- Further evidence of a weak fiber-matrix interface and early debonding of the untreated specimens was provided by the higher attenuation of ultrasonic waves, due to increased scattering by internal defects and as residual cooling stresses produced a density of transverse microcracks ten times as high as in the laminates manufactured with thermally treated fibers
- The improved fiber-matrix interface of the desized laminates is best appreciated through the macroscopic mechanical properties. When these specimens were subjected to tensile tests with the reinforcement at ±45${}^{\circ }$ with respect to the loading axis and compared with the untreated ones, strength increased by 37%, strain to failure by 190% and energy per unit volume absorbed by 230% ( 0.77 MJ/m${}^{3}$ vs. 2.54 MJ/m${}^{3}$ )

As a final consideration, these results showed that through a relatively simple modification of commercial carbon fibers, a CF/PEEK composite can achieve mechanical properties on par with thermosetting composites. However, given that the usage of thermoplastic matrices still presents some technological issues an additional effort should be made to meet the safety and durability levels demanded by industrial applications. In this regard, a careful evaluation of the interface properties deserves special attention due to its influence on the mechanical behavior of the composite and should be carried out in every experimental campaign that entails the characterization of thermoplastic matrix composites.

## Figures and Tables

**Table 1:** Comparison of atomic concentrations and O/C ratio on fibers.

Type of Fiber	C (%)	O (%)	N (%)	O/C
*0-CFs* (untreated)	78.4	20.5	1.1	0.26
*I-CFs* (desized)	79.5	19.8	0.7	0.25

**Table 2:** Summary of results from tensile tests for the as-received and treated carbon fibers: failure load $F_{b}$ and Weibull parameters α and β.

Type of Fiber	$F_{b}$ [N]	α	β [N]
*0-CFs* (untreated)	0.14 ± 0.03	5.26	0.140
*I-CFs* (desized)	0.13 ± 0.03	5.42	0.142

**Table 3:** Elastic modulus and hardness for neat matrix, untreated and treated laminates measured through nanoindentation.

Material	*E* [GPa]	*H* [GPa]
Neat matrix	4.4 ± 0.1	0.28 ± 0.01
*Lam${}_{0}$* matrix	6.3 ± 0.2	0.44 ± 0.02
*Lam${}_{I}$* matrix	5.6 ± 0.2	0.33 ± 0.01

**Table 4:** Fiber-matrix interface parameters extracted from the push-in tests.

Laminate Interface	$r_{f}$ [μm]	$P_{c}$ [mN]	$S_{o}$ [N/mm]	$\tau_{c}$ [MPa]
*Lam${}_{0}$*	3.6 ± 0.2	26 ± 5	177 ± 12	26 ± 5
*Lam${}_{I}$*	3.6 ± 0.2	41 ± 4	170 ± 4	33 ± 3

**Table 5:** Tensile elastic modulus (*E*), strength ($\sigma_{f}$) and strain to failure ($\epsilon_{f}$) for different materials (*Lam${}_{0}$*, *Lam${}_{I}$*) and fiber orientations (0${}^{\circ }$/90${}^{\circ }$ and ±45${}^{\circ }$).

Material	*E* [GPa]	$\sigma_{f}$ [MPa]	$\epsilon_{f}$ [%]
*Lam${}_{0}$* 0${}^{\circ }$/90${}^{\circ }$	63 ± 5	640 ± 30	1.01 ± 0.15
*Lam${}_{I}$* 0${}^{\circ }$/90${}^{\circ }$	69 ± 3	690 ± 30	0.99 ± 0.12
*Lam${}_{0}$*±45${}^{\circ }$	19 ± 2	144 ± 14	2.0 ± 0.2
*Lam${}_{I}$*±45${}^{\circ }$	20 ± 2	197 ± 9	5.8 ± 0.3

## Data Availability

Not applicable.

## References

[1] Ribeiro F.R.C., Modolo R.C.E., Kulakowski M.P., Brehm F.A., Moraes C.A.M., Ferreira V.M., Mesquita E.F.T., de Azevedo A.R.G., Monteiro S.N. (2022). Production of belite based clinker from ornamental stone processing sludge and calcium carbonate sludge with lower CO_2_ emissions. Materials.

[2] Zhao Q., An L., Li C., Zhang L., Jiang J., Li Y. (2022). Environment-friendly recycling of CFRP composites via gentle solvent system at atmospheric pressure. Compos. Sci. Technol..

[3] Fernández A., Santangelo-Muro M., Fernández-Blázquez J.P., Lopes C.S., Molina-Aldareguia J.M. (2021). Processing and properties of long recycled-carbon-fibre reinforced polypropylene. Compos. Part B Eng..

[4] Asmatulu E., Twomey J., Overcash M. (2014). Recycling of fiber-reinforced composites and direct structural composite recycling concept. J. Compos. Mater..

[5] Yang Y., Boom R., Irion B., van Heerden D.J., Kuiper P., de Wit H. (2012). Recycling of composite materials. Chem. Eng. Process. Process Intensif..

[6] Díez-Pascual A.M., Naffakh M., González-Domínguez J.M., Ansón A., Martínez-Rubi Y., Martínez M.T., Simard B., Gómez M.A. (2010). High performance PEEK/carbon nanotube composites compatibilized with polysulfones-I. Structure and thermal properties. Carbon.

[7] Diez-Pascual A.M., Ashrafi B., Naffakh M., González-Domínguez J.M., Johnston A., Simard B., Martinez M.T., Gómez-Fatou M.A. (2011). Influence of carbon nanotubes on the thermal, electrical and mechanical properties of poly (ether ether ketone)/glass fiber laminates. Carbon.

[8] Zhang Y., Tao W., Zhang Y., Tang L., Gu J., Jiang Z. (2018). Continuous carbon fiber/crosslinkable poly (ether ether ketone) laminated composites with outstanding mechanical properties, robust solvent resistance and excellent thermal stability. Compos. Sci. Technol..

[9] Panda S., Sarangi M., Chowdhury S.R. (2019). Examinations on PEEK wear debris accumulation over counter surfaces in room and vacuum sliding environments. Polym. Test..

[10] Tardif X., Pignon B., Boyard N., Schmelzer J.W., Sobotka V., Delaunay D., Schick C. (2014). Experimental study of crystallization of PolyEtherEtherKetone (PEEK) over a large temperature range using a nano-calorimeter. Polym. Test..

[11] Rattan R., Bijwe J. (2006). Carbon fabric reinforced polyetherimide composites: Influence of weave of fabric and processing parameters on performance properties and erosive wear. Mater. Sci. Eng. A.

[12] Ivanov S.G., Beyens D., Gorbatikh L., Lomov S.V. (2017). Damage development in woven carbon fibre thermoplastic laminates with PPS and PEEK matrices: A comparative study. J. Compos. Mater..

[13] Shekar R.I., Kotresh T., Rao P.D., Kumar K. (2009). Properties of high modulus PEEK yarns for aerospace applications. J. Appl. Polym. Sci..

[14] Gao X., Huang Z., Zhou H., Li D., Li Y., Wang Y. (2019). Higher mechanical performances of CF/PEEK composite laminates via reducing interlayer porosity based on the affinity of functional s-PEEK. Polym. Compos..

[15] Pukanszky B. (1990). Influence of interface interaction on the ultimate tensile properties of polymer composites. Composites.

[16] Blassiau S., Thionnet A., Bunsell A.R. (2009). Three-dimensional analysis of load transfer micro-mechanisms in fibre/matrix composites. Compos. Sci. Technol..

[17] Rosen B.W. (1964). Tensile failure of fibrous composites. AIAA J..

[18] Qiu B., Sun T., Li M., Chen Y., Zhou S., Liang M., Zou H. (2020). High micromechanical interlocking graphene oxide/carboxymethyl cellulose composite architectures for enhancing the interface adhesion between carbon fiber and epoxy. Compos. Part A Appl. Sci. Manuf..

[19] Yang L., Thomason J. (2012). Development and application of micromechanical techniques for characterising interfacial shear strength in fibre-thermoplastic composites. Polym. Test..

[20] Zhou X.F., Wagner H., Nutt S. (2001). Interfacial properties of polymer composites measured by push-out and fragmentation tests. Compos. Part A Appl. Sci. Manuf..

[21] Chandra N., Ghonem H. (2001). Interfacial mechanics of push-out tests: Theory and experiments. Compos. Part A Appl. Sci. Manuf..

[22] Naya F., Molina-Aldareguia J., Lopes C., González C., LLorca J. (2017). Interface characterization in fiber-reinforced polymer–matrix composites. JOM.

[23] Rodríguez M., Molina-Aldareguía J.M., González C., LLorca J. (2012). A methodology to measure the interface shear strength by means of the fiber push-in test. Compos. Sci. Technol..

[24] Medina M C., Molina-Aldareguía J.M., González C., Melendrez M.F., Flores P., LLorca J. (2016). Comparison of push-in and push-out tests for measuring interfacial shear strength in nano-reinforced composite materials. J. Compos. Mater..

[25] Hassan E.A., Elagib T.H., Memon H., Yu M., Zhu S. (2019). Surface modification of carbon fibers by grafting peek-nh2 for improving interfacial adhesion with polyetheretherketone. Materials.

[26] Yuan C., Li D., Yuan X., Liu L., Huang Y. (2021). Preparation of semi-aliphatic polyimide for organic-solvent-free sizing agent in CF/PEEK composites. Compos. Sci. Technol..

[27] Ren T., Zhu G., Hou X., Li B., Hao Y. (2021). Improvement of interfacial interactions in CF/PEEK composites by an s-PSF/graphene oxide compound sizing agent. J. Appl. Polym. Sci..

[28] Lyu H., Jiang N., Hu J., Li Y., Zhou N., Zhang D. (2022). Preparing water-based phosphorylated PEEK sizing agent for CF/PEEK interface enhancement. Compos. Sci. Technol..

[29] Hassan E.A., Yang L., Elagib T.H., Ge D., Lv X., Zhou J., Yu M., Zhu S. (2019). Synergistic effect of hydrogen bonding and *π*-*π* stacking in interface of CF/PEEK composites. Compos. Part B Eng..

[30] Hassan E.A., Ge D., Zhu S., Yang L., Zhou J., Yu M. (2019). Enhancing CF/PEEK composites by CF decoration with polyimide and loosely-packed CNT arrays. Compos. Part A Appl. Sci. Manuf..

[31] Lyu H., Jiang N., Li Y., Zhang D. (2021). Enhancing CF/PEEK interfacial adhesion by modified PEEK grafted with carbon nanotubes. Compos. Sci. Technol..

[32] Silvestro L., Ruviaro A., Lima G., de Matos P., de Azevedo A.R., Monteiro S.N., Gleize P. (2021). Influence of ultrasonication of functionalized carbon nanotubes on the rheology, hydration, and compressive strength of portland cement pastes. Materials.

[33] Jongvivatsakul P., Thongchom C., Mathuros A., Prasertsri T., Adamu M., Orasutthikul S., Lenwari A., Charainpanitkul T. (2022). Enhancing bonding behavior between carbon fiber-reinforced polymer plates and concrete using carbon nanotube reinforced epoxy composites. Case Stud. Constr. Mater..

[34] Dai Z., Shi F., Zhang B., Li M., Zhang Z. (2011). Effect of sizing on carbon fiber surface properties and fibers/epoxy interfacial adhesion. Appl. Surf. Sci..

[35] Li N., Chen J., Liu H., Dong A., Wang K., Zhao Y. (2019). Effect of preheat treatment on carbon fiber surface properties and fiber/PEEK interfacial behavior. Polym. Compos..

[36] Wenbo L., Shu Z., Lifeng H., Weicheng J., Fan Y., Xiaofei L., Rongguo W. (2013). Interfacial shear strength in carbon fiber-reinforced poly (phthalazinone ether ketone) composites. Polym. Compos..

[37] Denault J., Vu-Khanh T. (1993). Fiber/matrix interaction in carbon/PEEK composites. J. Thermoplast. Compos. Mater..

[38] Voyiadjis G.Z., Samadi-Dooki A., Malekmotiei L. (2017). Nanoindentation of high performance semicrystalline polymers: A case study on PEEK. Polym. Test..

[39] Hardiman M., Vaughan T., McCarthy C. (2012). The effect of fibre constraint in the nanoindentation of fibrous composite microstructures: A finite element investigation. Comput. Mater. Sci..

[40] Oliver W.C., Pharr G.M. (2004). Measurement of hardness and elastic modulus by instrumented indentation: Advances in understanding and refinements to methodology. J. Mater. Res..

[41] Cox H. (1952). The elasticity and strength of paper and other fibrous materials. Br. J. Appl. Phys..

[42] Molina-Aldareguía J.M., Rodríguez M., González C., LLorca J. (2011). An experimental and numerical study of the influence of local effects on the application of the fibre push-in test. Philos. Mag..

[43] Okabe Y., Mizutani T., Yashiro S., Takeda N. (2002). Detection of microscopic damages in composite laminates. Compos. Sci. Technol..

[44] (2008). Standard tEst Method for Tensile Properties of Polymer Matrix Composite Materials.

[45] Nordström Y., Joffe R., Sjöholm E. (2013). Mechanical characterization and application of Weibull statistics to the strength of softwood lignin-based carbon fibers. J. Appl. Polym. Sci..

[46] Iqbal T., Briscoe B.J., Yasin S., Luckham P.F. (2013). Nanoindentation response of poly (ether ether ketone) surfaces—A semicrystalline bimodal behavior. J. Appl. Polym. Sci..

[47] Gao S., Gao S., Xu B., Yu H. (2015). Effects of different pH-values on the nanomechanical surface properties of PEEK and CFR-PEEK compared to dental resin-based materials. Materials.

[48] Parlevliet P.P., Bersee H.E., Beukers A. (2006). Residual stresses in thermoplastic composites—A study of the literature—Part I: Formation of residual stresses. Compos. Part A Appl. Sci. Manuf..

[49] Jeronimidis G., Parkyn A. (1988). Residual stresses in carbon fibre-thermoplastic matrix laminates. J. Compos. Mater..

[50] Yang L., Yan Y., Ma J., Liu B. (2013). Effects of inter-fiber spacing and thermal residual stress on transverse failure of fiber-reinforced polymer–matrix composites. Comput. Mater. Sci..

[51] Gentz M., Benedikt B., Sutter J., Kumosa M. (2004). Residual stresses in unidirectional graphite fiber/polyimide composites as a function of aging. Compos. Sci. Technol..

[52] Parlevliet P.P., Bersee H.E., Beukers A. (2007). Residual stresses in thermoplastic composites–a study of the literature. Part III: Effects of thermal residual stresses. Compos. Part A Appl. Sci. Manuf..

[53] Kas Y.O., Kaynak C. (2005). Ultrasonic (C-scan) and microscopic evaluation of resin transfer molded epoxy composite plates. Polym. Test..

[54] Hsu D. (2013). Non-destructive evaluation (NDE) of aerospace composites: Ultrasonic techniques. Non-Destructive Evaluation (NDE) of Polymer Matrix Composites.

[55] Jian L. (2021). Effect of sizing agent on interfacial properties of carbon fiber-reinforced PMMA composite. Compos. Adv. Mater..

[56] Hao S., He L., Liu J., Liu Y., Rudd C., Liu X. (2021). Recovery of carbon fibre from waste prepreg via microwave pyrolysis. Polymers.

[57] Giraud I., Franceschi-Messant S., Perez E., Lacabanne C., Dantras E. (2013). Preparation of aqueous dispersion of thermoplastic sizing agent for carbon fiber by emulsion/solvent evaporation. Appl. Surf. Sci..

[58] Stier B., Simon J.W., Reese S. (2015). Comparing experimental results to a numerical meso-scale approach for woven fiber reinforced plastics. Compos. Struct..

[59] Daggumati S., De Baere I., Van Paepegem W., Degrieck J., Xu J., Lomov S.V., Verpoest I. (2010). Local damage in a 5-harness satin weave composite under static tension: Part I–Experimental analysis. Compos. Sci. Technol..

[60] Gliesche K., Hübner T., Orawetz H. (2005). Investigations of in-plane shear properties of ±45-carbon/epoxy composites using tensile testing and optical deformation analysis. Compos. Sci. Technol..

[61] Bergmann T., Heimbs S., Maier M. (2015). Mechanical properties and energy absorption capability of woven fabric composites under ±45 off-axis tension. Compos. Struct..

